# Territory Occupancy and Parental Quality as Proxies for Spatial Prioritization of Conservation Areas

**DOI:** 10.1371/journal.pone.0097679

**Published:** 2014-05-16

**Authors:** Matthias Tschumi, Michael Schaub, Raphaël Arlettaz

**Affiliations:** 1 Division of Conservation Biology, Institute of Ecology and Evolution, University of Bern, Bern, Switzerland; 2 Swiss Ornithological Institute, Sempach, Switzerland; 3 Swiss Ornithological Institute, Valais Field Station, Sion, Switzerland; University of Lleida, Spain

## Abstract

In order to maximize their fitness, individuals aim at choosing territories offering the most appropriate combination of resources. As population size fluctuates in time, the frequency of breeding territory occupancy reflects territory quality. We investigated the relationships between the frequency of territory occupancy (2002–2009) vs. habitat characteristics, prey abundance, reproductive success and parental traits in hoopoes *Upupa epops* L., with the objective to define proxies for the delineation of conservation priority areas. We predicted that the distribution of phenotypes is despotic and sought for phenotypic characteristics expressing dominance. Our findings support the hypothesis of a despotic distribution. Territory selection was non-random: frequently occupied territories were settled earlier in the season and yielded higher annual reproductive success, but the frequency of territory occupancy could not be related to any habitat characteristics. Males found in frequently occupied territories showed traits expressing dominance (i.e. larger body size and mass, and older age). In contrast, morphological traits of females were not related to the frequency of territory occupancy, suggesting that territory selection and maintenance were essentially a male's task. Settlement time in spring, reproductive success achieved in a given territory, as well as phenotypic traits and age of male territory holders reflected territory quality, providing good proxies for assessing priority areas for conservation management.

## Introduction

Territory choice has a crucial impact on individual fitness since the acquisition of a highly suitable territory is essential for survival and successful reproduction [1], [2]. Individuals should settle in the highest quality territories to maximize their fitness. High quality territories are those that contain an optimal combination of essential resources needed for reproduction and survival, such as nest-sites, food, and concealment from predators [3]. If individuals can move between territories, the ideal free distribution model (IFD) predicts that they should occupy habitat patches in proportion to the amount of available resources [4], [5]. Thus, in areas of high habitat quality, territories are expected to be smaller than those in areas of lower quality habitats. Due to a roughly similar amount of resources available per territory this should result in equivalent reproductive success among territories across the area, insofar as an IFD model applies.

The IFD model assumes that all individuals have the same competitive abilities, an assumption which is often violated [6]. The ideal despotic distribution model (IDD), on the other hand, takes the variations in competitive abilities among individuals into account and thus predicts that stronger, rather than weaker, individuals settle in higher quality territories [4], [5]. Under an IDD scenario, reproductive success varies spatially, with greater success in territories of high quality. Both models postulate that the distribution of breeders in a heterogeneous environment is non-random in space and time: high quality territories tend to be occupied when population density is low, whereas low quality territories are only occupied during high population density. Thus, the number of times a territory is occupied over a given period of time (i.e. its occupancy rate) can be used as a reliable measure for territory quality [1], [7]–[9].

Using territory occupancy frequency as a measure of territory quality provides a means of identifying the key habitat factors determining quality [1], [10]. These factors can include any resources such as food supply, nesting sites and/or structural habitat variables. The identification of these key factors can constitute an essential step in species' conservation and management.

Migratory bird species have to select a breeding territory every year. Typically, older individuals arrive first at the breeding grounds [11], [12] and can therefore freely select their territory. In addition, early arriving individuals are often in better physiological condition than those arriving later [13]. The outcome of this settlement process is that the best territories are typically occupied by the most dominant, i.e. highest quality, individuals [14].

The competitive ability of an individual is difficult to assess, and usually phenotypic traits, such as age, body size or mass are considered. Older individuals often dominate younger individuals, since they are more experienced [2]. The same holds for larger and/or heavier individuals that are physically stronger [3], [15]. Since individuals with high competitive abilities (dominant individuals) choose territories of higher quality, the frequency of territory occupancy often correlates positively with phenotypic traits expressing the dominance of territory occupants [14].

Few studies have investigated the links between territory occupancy, breeding success, environmental characteristics, and parental phenotypic traits [16]. We studied the correlates and determinants of territory selection of hoopoes (*Upupa epops* L.) in southwestern Switzerland. Using the frequency of territory occupancy during 8 years as an indication of territory quality, we studied the relationships between territory occupancy, habitat parameters within territories, and individual characteristics of territory occupants at two spatial scales, and then evaluated the consequences of territory occupancy and individual qualities on reproductive success. Previous studies of the same population have shown that hoopoes mainly feed on mole crickets (*Gryllotalpa gryllotalpa* L.) [17], [18], which are sought for amongst sparse ground vegetation [19]. We therefore predicted a positive relationship between frequency of territory occupancy vs. mole cricket occurrence and amount of bare soil, respectively. Secondly, we tested whether phenotypic traits of territory occupants were related to frequency of territory occupancy, predicting that larger, heavier and older individuals occupy territories that typically have a higher occupancy rate. Finally, we tested whether reproductive success was related to territory occupancy or to the phenotypic traits of territory occupants, in both cases anticipating positive relationships. Our ultimate goal was to establish more straightforward cues to assess territory quality other than occupancy and thus to spatially identify priority areas for efficient conservation management.

## Methods

### Study species

Hoopoes preferably inhabit semi-open, dry and sunny areas of southern Europe, north-western Africa and central Asia. Typical breeding habitats often include traditionally cultivated areas in central Europe [18], but high-intensity farmland can also be inhabited provided that essential resources are available [17], [19]. Hoopoes are eclectic secondary cavity breeders, occupying hollow trees and walls, as well as nest boxes. Hoopoes often raise two broods a year [17]. After World War II hoopoes have undergone large declines in central Europe, including Switzerland. The species is considered to be vulnerable in Switzerland [20], although populations have been increasing recently [21].

### Study area

The study was conducted on the plain of the upper Rhône valley (Valais, 46.2°N, 7.4°E; 480 m above sea level) from April to August 2002–2009. The study area covered 62 km^2^, dominated by industrial farming, mainly consisting of fruit-tree plantations, vineyards and vegetable crops. For a more detailed description of the region see [21]. In the study area, 690 nest boxes designed for hoopoes (dimension 20×20×30 cm; entrance hole diameter: 55 mm) were installed gradually from 1997 to winter 2002 at 367 locations, with usually two nest boxes installed in a given building. The focal hoopoe population responded rapidly to the installation of nest boxes, with a ca. 2-fold increase in population size within a few years during the study period [21].

### Sampling design

#### Ethics statement

All sites were visited with landowner permission. All research protocols involving experimental activities with birds were approved by the Swiss Federal Office for the Environment (FOEN) and the Swiss Ornithological Institute and comply with the Swiss legislation.

#### Territory occupancy

To determine territory occupancy we inspected nest boxes every second week throughout the breeding season. Every year the inspections started before the first nest boxes were expected to be occupied (i.e. end of March). All occupied nest boxes were thereafter checked every third day to collect information about clutch size, number of fledglings, and phenology. For each of the 300 locations we added up the number of years in which they were occupied by breeding hoopoes, hereafter designated ‘territory occupancy’ (range 0–8 years, from 2002–2009). We defined a territory to be occupied in a year if at least one egg was laid in it in that year. Double occupancies of a territory within a given year were not considered because, usually, at the time when second broods are initiated, many nest boxes are already occupied and consequently territory choice is already much restricted.

#### Individual phenotypic traits

The majority of breeding hoopoes were caught using mist-nets or spring traps placed at the nest box entrance; females were also taken by hand from the nest box while brooding. Capture attempts started 4 days after the hatching of the first chick; they ended after fledging or after a maximum effort of 12.5 h for capture. The same standardized design was used every year. We determined sex and age (two classes: one year old and older, recognizable from moult patterns of wing feathers; Mosimann-Kampe unpublished data), and measured the lengths of bill, crest, the fifth primary feather (P5), the first tail feather (R1) and tarsus, as well as body mass. All captured individuals as well as all nestlings were ringed [22].

#### Habitat mapping

From a total of 367 available breeding locations, only those that were occupied at least once by a breeding hoopoe between 2002 and 2008 were retained (n = 172). 100 of them were chosen randomly, the habitat of which was mapped in 2009. A circle with a 300 m radius (corresponding approximately to a mean home range size of 40 ha [23]) was drawn around each of the 100 nest box locations. This circular area was considered to be a territory. Various habitat variables (see Table 1 and Table S1 for descriptive statistics) were recorded at 30 locations randomly selected within this circular territory in order to describe habitat features. In addition, we used a soil penetrometer to measure soil density at all sampling points. To correct for the seasonal changes in soil density, we measured the soil resistance once a week (n = 15) between April and August at a single location in the middle of the study area (46°13′03″N 07°20′41″E). The chosen location showed average soil conditions comparable to the main conditions prevailing at most of the other sampling points. Five measures each were taken at every sampling occasion. In addition, we used ground water table maps retrieved from publicly available data (Département des Transports de l'Equipement et de l'Environnement, Etat du Valais & Centre de Recherche sur l'Environnement Alpin – CREALP) to look at the link between mole cricket occurrence and ground water depth.

**Table 1:** Description of habitat variables recorded at mapping locations for modeling hoopoe territory occupancy and mole cricket occupancy.

Parameter	Levels	Description
Habitat type	apple tree plantation	
	apricot tree plantation	
	pear tree plantation	
	arable field	
	vineyard	
	grassland	
	river bank	
	wood	
	non-tarred road	
	unsuitable area	building, tarred road, open water
Vegetation cover	-	continuous (to nearest 10%)
Mowing	yes or no	regular mowing of the driving track (only for fruit tree plantations)
Ground management	mowing	management of the vegetation strip underneath plantation trees (only for fruit tree plantations)
	herbicide application	strip underneath plantation trees (only for fruit tree plantations)
	mechanical veg. removal	trees (only for fruit tree plantations)
	no treatment	plantations)
Soil type	1) silty soil with no-till-limited presence of sand	characterisation of top soil layer
	2) silty soil with obvious presence of sand	
	3) silty soil embedded in a matrix dominated by gravel, stones or pebbles	
	4) sandy soil where large structures such as gravel and pebbles are absent	
	5) sand embedded in a matrix dominated by gravel, stones or pebbles	
	6) all kind of humus rich soil (decomposed litter)	
Soil density	-	continuous (0–15 in steps of 0.5) Five measures each at every sampling occasion using a soil penetrometer

#### Mole cricket occurrence

To assess the relationship between mole cricket occurrence and habitat factors we sampled detection/non-detection data of mole cricket traces at 97 plots, along with abiotic and biotic habitat factors (depth of ground water table, soil density, soil type, vegetation cover) in 2009. These plots were randomly selected using ArcGIS 9.x [24] within the same area where all other habitat variables were collected (see above), but exclusively within fruit tree plantations, because this is the favoured foraging habitat of hoopoes [23]. In order to ensure sufficient contrast, we used a stratified sampling design: plots were chosen at random in areas known to have a high and low ground water table, respectively (10 in each). Another 10 plots were randomly selected within areas with very gravelly soil. The remaining 67 plots were chosen randomly from the remaining areas that showed average soil type and ground water table conditions. The presence of mole crickets was determined by searching, during 10 min, for underground galleries and holes at the soil surface along three 10 m transects separated by three tree rows. If at least one gallery or entrance hole was found, mole cricket presence was considered as detected at that visit. All plots were sampled four times in June, during the peak period of mole cricket activity [25]. We recorded habitat covariates we thought were relevant for mole cricket occurrence (Table 1), but also took into account covariates that might have affected detectability, namely vegetation height and weather variables (precipitation on the day preceding a visit; daily average temperature; daily maximum temperature, and daily duration of sunshine; all obtained from the meteorological station at Sion: 46°13′06″N, 07°19′48″E).

### Statistical analyses

#### Random territory choice and effects on productivity

All statistical analyses, except estimations of mole cricket occupancy (see below), were conducted using the software R [26]. To test whether hoopoes selected breeding locations randomly, we compared the frequency distribution of the observed occupancy of all territories that were occupied at least once from 2002–2009 (n = 192) with the frequency distribution of occupancy that would be predicted in a scenario of random selection of territories. We used a simulation approach to determine the frequency distribution of occupancy under random territory selection that takes into account the variable number of breeding pairs. Specifically, we randomly selected for each year from the list of the available territories the number of territories that corresponds to the number of breeding pairs in a given year and assigned them as occupied. We then tabulated occupancy of each territory, derived the frequency distribution and repeated this procedure 1000 times. The mean frequency distribution was then taken as the reference for random territory selection. We compared the two frequency distributions with the Pearson's chi-square test.

To study the relationships between breeding phenology and reproductive success vs. territory occupancy we used linear mixed effects models. We used the hatching date of chicks as a proxy for the date of territory settlement and then modelled it using territory identity as a random effect, and the year of territory occupancy as a fixed effect. The inclusion of territory identity enabled us to include modelling of the hatching dates for every year, thus avoiding pseudo-replication. Reproductive success represented by the total number of fledglings per territory and year was modelled as a function of territory occupancy (i.e. number of times the territory was occupied between 2002 and 2009) using territory identity as a random effect and assuming a Poisson error distribution. Furthermore, we tested whether the probability of second broods was related to the occupancy of territories. For this purpose we modelled the binary variable indicating the presence or absence of a second brood in every year and territory with territory occupancy, using territory identity as a random effect. All these models were compared to corresponding models without the variable ‘territory occupancy’, and model averaged parameter estimates were calculated based on AIC weights (Akaike's Information Criterion (AIC) [27]).

#### Individual phenotypic traits

To evaluate whether phenotypic traits of territory occupants were related to territory occupancy, we fitted linear mixed effects models (normal error distribution) with different morphological traits (bill length, crest length, length of P5 and first tail feather R1, tarsus length, body mass) as response variables, and territory occupancy as an explanatory variable. Again, territory identity was included as a random effect. These models were evaluated for males and females separately given the existence of a slight sexual dimorphism (males: n = 626; females: n = 758). As before, we compared the models with corresponding models without the variable ‘territory occupancy’ and used model averaged parameter estimates based on AIC weights for inference.

Secondly, we tested whether the age of territory occupants (two categories: one year old and older) was related to territory occupancy. Linear mixed effect models with a binomial error distribution and territory identity as a random effect were used to model the binary response variable age. Modelling was performed separately for males (n = 553) and females (n = 654). The resulting models were again compared with a model without the variable ‘territory occupancy’, and model averaged parameter estimates based on AIC weights were used for inference.

To test whether the number of fledglings was linked to the morphology and age of breeding individuals we used linear mixed effects models with a Poisson error distribution. The number of fledglings of a given breeding adult in a year was modelled using its different morphological traits and age as explanatory variables, and the individual identity as a random effect. Confounding factors such as the year and date of the current brood, and whether it was a first or second brood were included in all models. The various models fitted were predefined in a sensible way and included either morphological traits and age separately, single morphological traits in combination with age, all morphological traits plus age, or none of these effects. Modelling was again performed for males (n = 397) and females (n = 434) separately. We computed model-averaged parameter estimates based on AIC weights.

#### Mole cricket occurrence

Mole cricket detection/non-detection data was analysed using occupancy models [28] with software MARK [29]. We used average daily temperature, maximum daily temperature, amount of rainfall, duration of sunshine, sampling event, vegetation cover, and vegetation height to model detection probability (p). To model occupancy probability (Ψ) we used depth of the ground water table, soil density (corrected for date, see above), soil type and vegetation cover as well as the quadratic terms of ground water table depth, soil density and vegetation cover, and two interactions, namely soil type*ground water table and soil type*soil density. We performed modelling in four steps. In a first step we used the full model for occupancy including all quadratic terms and interactions and explored the effect of different combinations of covariates for detection probability. In steps two and three we investigated the importance of different quadratic terms and interactions, respectively, on occupancy. Finally we evaluated the remaining combinations of variables that were neither involved in a quadratic term nor in an interaction on occupancy. The models were ranked in every step using AICc values (AIC corrected for small sample size [27]), and the best models (ΔAICc ≤ 2) were then selected for the next step. To get the best parameter estimates for the extrapolation of mole cricket occurrence across the study area we performed model averaging, subsequently using the best models of the final step, i.e. those models for which the sum of AICc weights was ≥ 0.9.

#### Relation of territory occupancy to habitat characteristics

To correct for seasonal changes of soil density we fitted a linear mixed effects model with soil density of the reference location (see above) as a response variable, date as an explanatory fixed variable, and visit as a random effect. This model revealed that soil density increased during the course of the season. Using the parameter estimates of this model we corrected all soil density measures for date.

Using the recorded habitat variables, and extrapolated information about mole cricket occurrence (see above) we modelled territory occupancy (binomial data) of hoopoes in the randomly selected 100 territories using mixed effects models (territory identity number as a random effect, habitat variables, and the predicted probability of occurrence of mole crickets, as fixed). The inclusion of territory identity as a random effect accounted for repeated measurements at territories, as each territory was delineated by up to 30 sampling points. We used the model-averaged parameter estimates obtained from the mole cricket occupancy modelling (see below), and point-specific habitat covariates to calculate the probability of mole cricket occurrence.

We modelled territory occupancy in two main steps each using territory variables from two spatial scales (large scale  = 300 m and small scale: a subset of the large scale  = 200 m radius). First, we evaluated the effect of habitat type on territory occupancy. Since a habitat type could be assigned to all points, the sample size was 3000 for the large spatial scale and 1388 for the smaller spatial scale. Secondly, we modelled structural habitat variables. Since they could not be assessed at all points, sample size was lower (large scale: n = 2378; small scale: n = 1153). Structural variables included soil density (corrected for date, see above), depth of ground water table, vegetation cover, and soil type as well as the probability of mole cricket occurrence as a measure of food abundance. We fitted a null model with an intercept only, a full model that included all structural variables and models that included only one structural variable. We also evaluated different combinations of structural variables and models with quadratic effects for soil density and vegetation cover. As fruit tree plantations are the main local foraging habitat of hoopoes [23], structural variables in that habitat may be the main determinants of overall habitat quality. Therefore, we tested the same combinations of models but this time only with structural variables measured in fruit tree plantations (large scale: n = 1182; small scale: n = 634).

## Results

### Random territory choice and effects on productivity

**Figure 1:**
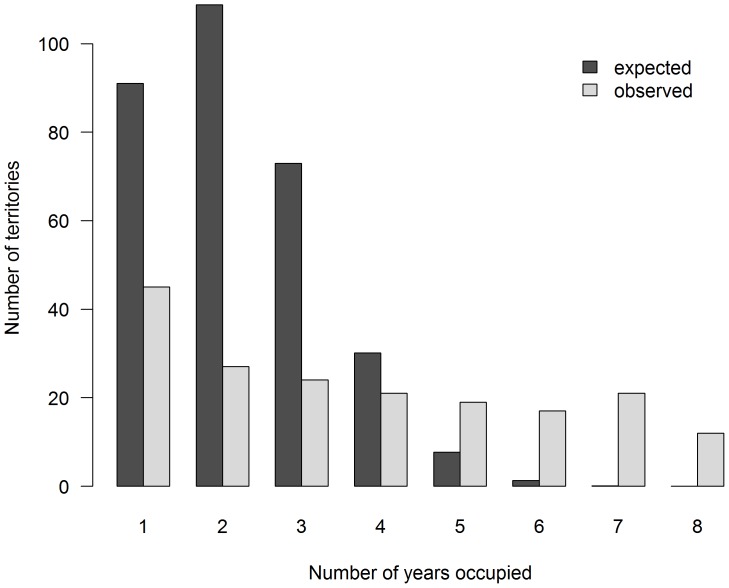
Observed and expected frequency of territory occupancy. The comparison of observed and expected (according to a random selection scenario) frequency of territory occupancy of hoopoes (2002–2009) shows the deviation from a random territory selection pattern (χ^2^ = 117.85, df = 7, *P*<0.001).

Hoopoes did not select territories at random (χ^2^ = 117.85, df = 7, *P*<0.001, Fig. 1). If territory selection had been random, we would have expected a greater number of territories to be occupied one to three times and a lower number of territories to be occupied more than four times than observed. The hatching date decreased noticeably with the frequency of territory occupancy (β = −2.44, SE = 0.39, AIC weight >0.99); thus, territories that were occupied more often were also occupied earlier in the season. The number of fledglings was also positively related with the frequency of territory occupancy (β = 0.07, SE = 0.01, territory random effect (variance)  = 0.05, AIC weight >0.99); thus more frequently occupied territories were overall more productive than less frequently occupied territories. The probability of rearing a second brood increased with the frequency of territory occupancy (β = 0.40, SE = 0.08, territory random effect (variance)  = 0.97, AIC weight >0.99), thus, the larger number of fledglings that were raised annually in high quality territories was mostly the result of a larger annual number of successfully reared broods.

### Individual phenotypic traits

The average age of breeders was strongly related to territory occupancy in males (β = 0.23, SE = 0.04, territory random effect (variance) <0.01, AIC weight >0.99), but not in females (β = 0.04, SE = 0.04, territory random effect (variance) <0.01, AIC weight  = 0.65). The relationship was positive in both sexes, thus older individuals tended to settle in territories that were more frequently occupied.

**Figure 2:**
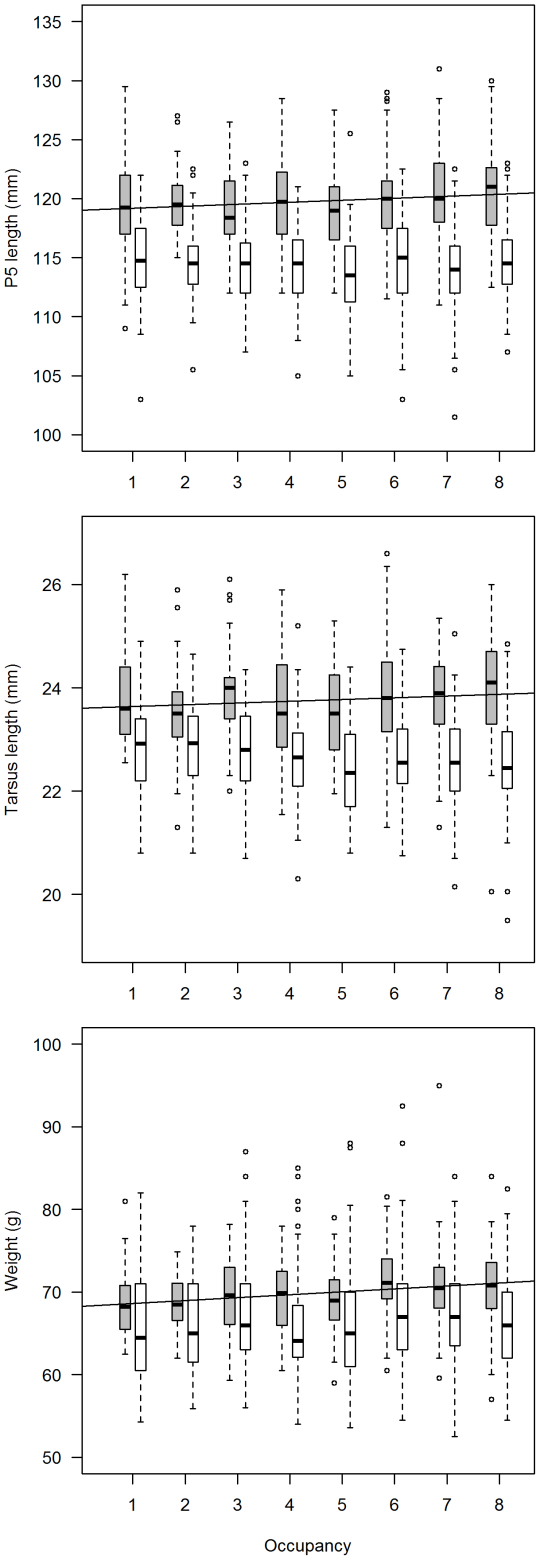
Relationship between territory occupancy and individual characteristics. Relationship between territory occupancy (2002–2009) vs. P5 (fifth primary feather) length, tarsus length and body mass of territory occupants. Males: grey bars; females: white bars. The regression lines refer to males for which trends were statistically significant.

There were positive relationships between territory occupancy and morphological characteristics in males, but not in females. In males, morphological traits increased with increasing territory occupancy, with three of them statistically supported: P5 length, tarsus length and body mass (Table 2). Thus, males present in territories of higher occupancy were larger and heavier than males present in rarely occupied territories (Fig. 2). By contrast, the null model was often supported in females, with only one morphological trait whose 95% confidence interval was non-overlapping with 0 (Table 2). This trait (tarsus length), however, had a negative estimate, indicating that small sized females would have a better chance to occupy a territory. There was no link between individual reproductive success and morphological traits and/or age. None of the models using morphological traits and/or age to model fledgling numbers was statistically supported (Table S2).

**Table 2:** Relationship between territory occupancy and morphological characteristics of territory occupants.

	Males					Females				
Morphological trait	Estimate	SE	*w*_i_	n	σ^2^	Estimate	SE	*w*_i_	n	σ^2^
Bill length	0.01	0.08	0.70	468	0.70	0.00	0.01	0.27	527	0.00
Crest length	0.00	0.02	0.27	578	0.36	0.00	0.02	0.27	641	0.36
P5 length	0.17	0.09	0.90	566	0.33	0.00	0.02	0.27	634	0.41
R1 length	0.02	0.05	0.34	551	0.78	−0.07	0.09	0.51	634	1.13
Tarsus length	0.03	0.02	0.82	577	0.03	−0.03	0.02	0.86	650	0.02
Body mass	0.35	0.09	1.00	609	1.51	0.03	0.06	0.33	735	0.00

Model averaged parameter estimates of the effect of territory occupancy on different morphological traits in male and female hoopoes, evaluated by linear mixed effects model. Given are the estimates, their standard errors (SE), the territory random effect variance (σ^2^), the AIC weights (*w*_i_), compared to the intercept model and sample size (n).

### Mole cricket occurrence

Mole cricket occupancy models with interactions (soil type*ground water table and soil type*soil density) were weakly supported by the data (ΔAICc = 13.72 compared to overall best; Table 3). The quadratic terms of soil density and ground water table seemed to be important and were included in the best 15 models. The averaged parameter estimates showed that the probability of detecting mole crickets decreased with increasing ground vegetation cover (β = −0.63; SE = 0.16), with a decreased duration of sunshine (β = 0.43; SE = 0.14), and varied slightly among sampling events.

**Table 3:** Mole cricket occupancy models.

Model	ΔAICc	*w*_i_	*K*	Deviance
Ψ (soilt+dens+gw+dens^2^) p (veg+sun)	0.00	0.46	11	401.15
Ψ (dens+gw+dens^2^) p (t+veg+sun)	3.35	0.09	10	407.05
Ψ (dens+dens^2^) p (t+veg+sun)	4.51	0.05	9	410.72
Ψ (soilt+dens+dens^2^) p (t+veg+sun)	4.79	0.04	13	400.62
Ψ (soilt+dens+veg+dens^2^) p (t+veg+sun)	5.01	0.04	14	398.09
Ψ (dens-veg+gw+dens^2^) p (t+veg+sun)	5.06	0.04	11	406.21
Ψ (dens+gw+dens^2^+gw^2^) p (t+veg+sun)	5.11	0.04	11	406.26
Ψ (dens+gw+dens^2^) p (veg+sun)	5.51	0.03	7	416.55
Ψ (dens+veg+dens^2^) p (t+veg+sun)	5.86	0.02	10	409.57
Ψ (soilt+dens+dens^2^) p (veg+sun)	6.31	0.02	10	410.02
Ψ (soilt+dens+veg+dens^2^) p (veg+sun)	6.45	0.02	11	407.60
Ψ (soilt+dens+gw+dens^2^+gw^2^) p (t+veg+sun)	6.46	0.02	15	396.71
Ψ (soilt+dens+veg+gw+dens^2^+gw^2^) p (t+veg+sun)	6.66	0.02	16	394.01
Ψ (soilt+dens+gw+dens^2^) p (t+veg+sun)	6.72	0.02	14	399.80
Ψ (dens+dens^2^) p (veg+sun)	6.75	0.02	6	420.12
Ψ (dens+veg+gw+dens^2^+gw^2^) p (t+veg+sun)	6.82	0.02	12	405.34
Ψ (dens+veg+gw+dens^2^) p (veg+sun)	7.07	0.01	8	415.73
Ψ (dens+gw+dens^2^+gw^2^) p (veg+sun)	7.08	0.01	8	415.74
Ψ (soilt+dens+veg+gw+dens^2^) p (t+veg+sun)	7.22	0.01	15	397.47
Ψ (soilt+dens+gw+dens^2^+gw^2^) p (veg+sun)	7.56	0.01	12	406.08
Ψ (soilt+dens+veg+gw+dens^2^+gw^2^) p (veg+sun)	7.67	0.01	13	403.51
Ψ (dens+veg+dens^2^) p (veg+sun)	8.00	0.01	7	419.04
Ψ (soilt+dens+veg+gw+dens^2^) p (veg+sun)	8.47	0.01	12	406.99
Ψ (dens+veg+gw+dens^2^+gw^2^) p (veg+sun)	8.64	0.01	9	414.85

Model selection summary for mole cricket occupancy (Ψ) and detection probability (p) in response to habitat parameters. Shown are the differences between the best and the current model (ΔAICc), the AIC weight of the current model (*w*_i_), the number of estimated parameters (*K*) and the deviance. The best 15 models (∑*w*_i_ = 0.9) are shown on top separated from the others by a horizontal line.

Covariates: dens  =  soil density, gw  =  height of ground water table, soilt  =  soil type, sun  =  daily sunshine duration, t =  sampling occasion, veg  =  vegetation cover, dens^2^  =  quadratic term for soil density, gw^2^  =  quadratic term for ground water table.

**Figure 3:**
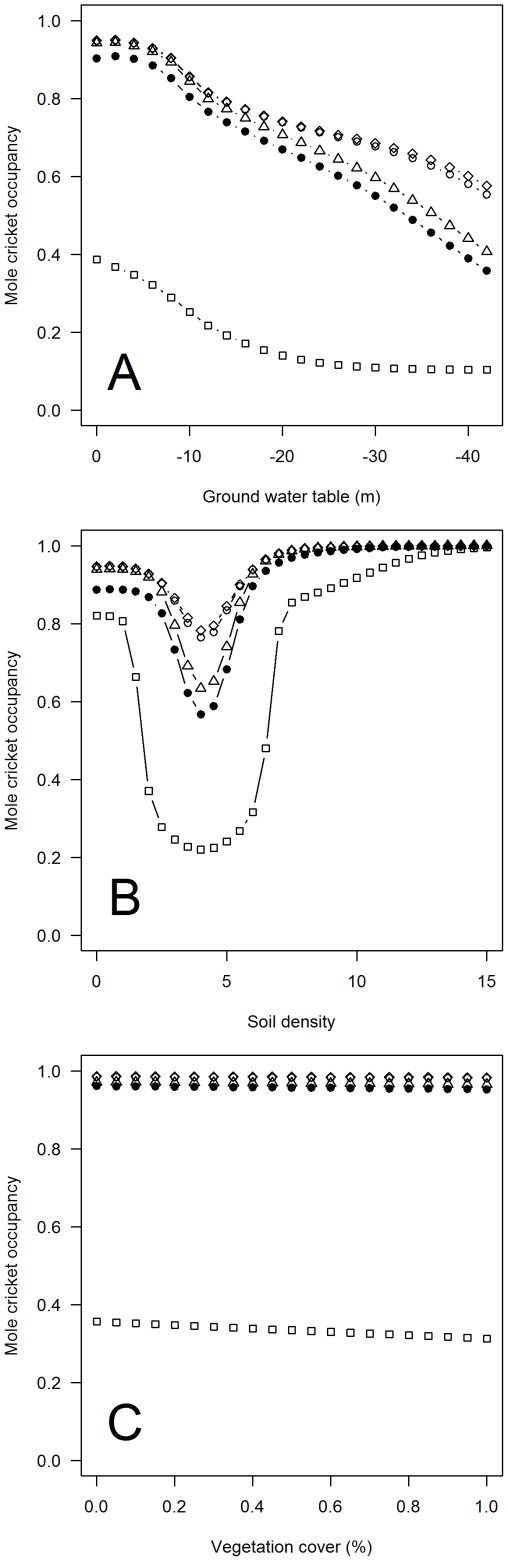
Occupancy probability of mole crickets in response to key habitat properties. Occupancy probability of mole crickets in response to a) depth of the ground water table, b) soil density, and c) surface vegetation cover. Model-averaged predictions are shown for five different soil types occurring at the sampling sites (Table 1). Closed circles represent soil type 1, open circles represent soil type 2, open squares represent soil type 3, open diamonds represent soil type 4 and open triangles represent soil type 5 (see Table 1 for the description of soil types).

The parameters most relevant for mole cricket occurrence were soil type, soil density, depth of ground water table and vegetation cover, as well as the same two above-mentioned quadratic terms (Fig. 3). It is striking that mole cricket occupancy was similar in all soil types except soil type 3 (silty soil embedded in a matrix dominated by gravel, stones or pebbles), in which mole crickets had a considerably lower probability of occurrence. Soil type 6 (soil rich in humus) did not occur in any sampled fruit tree plantation and was therefore not included. Mole cricket occurrence declined with increasing depth of the ground water table (Fig. 3). The relationship with soil density was more complex; mole cricket occurrence was higher in soft and hard soils compared to medium soils (Fig. 3). This was most pronounced with soil type 3. As expected, vegetation cover had only a weak impact on mole cricket occupancy (Fig. 3).

### Relation of territory occupancy and habitat characteristics

At both spatial scales, the simplest model including none of the habitat variables was the best, suggesting that the recorded habitat characteristics and mole cricket occurrence were not related to territory occupancy (Table 4). This was also true when only fruit tree plantations were considered (Table S3).

**Table 4:** Environmental determinants of territory occupancy.

	radius = 300 m					radius = 200 m				
Model	ΔAIC	*w*_i_	*K*	Deviance	σ^2^	ΔAIC	*w*_i_	*K*	Deviance	σ^2^
1) Evaluation of habitat type:										
intercept	0.00	0.99	2	6778.3	3.96	0.00	1.00	2	3401.3	3.32
habitat type	8.99	0.01	11	6763.3	3.96	15.35	0.00	11	3398.6	3.31
2) Evaluation of structural variables:										
intercept	0.00	0.40	2	5471.6	3.80	0.00	0.40	2	2891.6	3.21
mole crickets*	1.99	0.15	3	5471.6	3.80	1.97	0.15	3	2891.5	3.21
dens*	2.00	0.15	3	5471.6	3.80	1.98	0.15	3	2891.5	3.21
gw*	2.00	0.15	3	5471.6	3.80	2.00	0.15	3	2891.6	3.21
veg*	2.00	0.15	3	5471.6	3.80	2.00	0.15	3	2891.5	3.21
soilt*	9.99	0.00	7	5471.6	3.80	9.94	0.00	7	2891.5	3.21
dens+gw+veg+mole crickets+soilt*	17.97	0.00	11	5471.6	3.80	17.90	0.00	11	2891.4	3.20

Model selection summary of 1) the effects of habitat type on frequency of territory occupancy within a radius of 300 m (n = 3000) and 200 m (n = 1388) and 2) of the structural variables (radii: 300 m, n = 2378; 200 m, n = 1153). Shown are the differences between the best and the current model (ΔAIC), the AIC weight of the current model (*w*_i_), the number of estimated parameters (*K*), the model deviance and the territory random effect variance (σ^2^).

*Covariates: mole crickets  =  mole cricket occurrence probability, dens  =  soil density, gw  =  ground water table, veg  =  vegetation cover, soilt  =  soil type.

## Discussion

This study shows that hoopoes select their territories in a non-random manner, indicating spatial heterogeneity regarding the availability of crucial resources. In agreement with our expectations, territory settlement occurred earlier in the season and reproductive success was higher in frequently occupied territories. However, our models were unable to identify the environmental determinants of territory occupancy: neither habitat type, nor vegetation characteristics, nor mole cricket occurrence correlated with the frequency of territory occupancy. This may be due, at least in part, to some artifact linked to the method used; we shall return to this point later on.

In line with the predictions of the ideal despotic distribution model (IDD), male hoopoes breeding in often occupied territories were larger and heavier than birds breeding in territories that were rarely occupied. However, no such relationship was apparent in females. The demonstration that frequently occupied territories had a higher reproductive output corroborates the findings from other studies [1], [3], [7]–[9], [30]–[32]. Frequently occupied territories were more likely to support second broods, which was key to increased territory-specific reproductive success. The difference in reproductive output among territories can either originate from differences in territory quality per se, or from differences in the quality of territory occupants [33]. Since hoopoes are short-lived (annual survival probability is about 40%, [22]), different individuals have contributed to the high breeding success in the territories of high occupancy. This suggests that differences in territory quality were of importance. However, experiments are necessary to disentangle effects of territory and individual quality. The non-random selection of breeding territories with a clear role of male phenotypic traits suggests that a hierarchical system is operating. This pattern again conforms to the IDD model.

The relationships between territory occupancy and individual characteristics (age, size, body mass) differed between the genders. This suggests that males and females may rely on different criteria for operating territory selection and deciding about reproductive strategy. In multiple-brooding bird species it is commonly the task of the male to judge territory quality and try to monopolize the best available sites [11], while females evaluate the quality of males rather than the quality of territories [30]. The patterns observed in hoopoes seem to match this view. First, it is exclusively the task of males to secure favorable territories, as commonly observed in other species [11]. Males are also more philopatric to their breeding territory than females [34]. Altogether, this suggests that dominant males monopolize the best territories and avoid dispersal.

There are two mechanisms that could explain why frequently occupied territories were occupied earlier in the season by older and larger hoopoe males: first, older birds are usually more experienced and thus have a higher hierarchical status enabling them to outcompete younger individuals [2], [3], [7], [12], [15], [31], [35]. Secondly, older migrating birds are more experienced, usually arriving earlier on the breeding grounds than younger ones, and are therefore free to choose the best territories [2], [11], [12].

Hoopoe males in frequently occupied territories were larger and heavier than males present in rarely occupied territories. Theory predicts that dominant individuals will occupy high quality territories [4], and thus we can conclude that body size and body mass are important for defining the hierarchical status of hoopoe males. It is, however, not clear whether body size is directly beneficial in terms of agonistic conflicts for territory acquisition, or indirectly as a result of the earlier arrival date [2], [15]. First-arrived males may be more efficient migrators that benefit from precedence in territory settlement. In contrast, body size traits of females were not positively correlated with territory occupancy. As females lose body mass during incubation [36], a possibly existing relationship may have been blurred, since not all females could be captured at the same nesting stage. For the other female traits, however, analogous spurious findings are unlikely.

Contrary to our expectations, we did not find any connection between frequency of territory occupancy and habitat characteristics or the occurrence of the main prey, mole crickets. This held true both when considering two spatial scales, and when only the favorite foraging habitat type (fruit tree plantations) was considered. These results could be interpreted as if all territories were equal regarding habitat and prey availability. Indeed in monotonous human-made ecosystems like fruit tree plantations, the variation of some habitat features is small due to similar management practices (Table S1) and the quality of the nesting site per se might be more important than the seemingly homogenous habitat around the nesting sites. However, as foraging hoopoes showed a clear preference for certain habitat types [23], we rather suggest that it is more likely that extant differences were simply not detected via our approach. First, it can be argued that we did not consider the most relevant habitat variables. This is quite unlikely, yet, given that our habitat descriptors were chosen from fine-grained, radio-tracking information about habitat selection [23]. Neither can changes in the farmland matrix during the course of the study be inferred, because fruit tree plantations, predominant in the study area, have a low renewing rate. Secondly, a recent study has showed that the proportion of mole crickets in chick's diet actually increases with territory occupancy (Guillod, unpublished data), suggesting that the abundance of the locally most profitable prey does link to territory occupancy and is thus a prime determinant of territory quality. We might simply have failed to evidence such a link because we could not measure mole cricket abundance directly and had instead to use mole cricket occurrence as a measure of food availability, which probably does not well reflect local abundance. Furthermore, we predicted mole cricket occurrence indirectly, by using habitat variables, and any such extant link may have been blurred by too much variation around the signal. Finally, and perhaps most importantly, the true distribution pattern of mole crickets within hoopoe territories is likely to be very patchy, imposing additional difficulty for a proper estimation of both spatial distribution and local abundance. In effect, radio-tracking of foraging hoopoes indicate the existence of small areas where prey abundance is very high, forming mole cricket clusters [23]. Finally, our assumption that hoopoe territories are circular, with the breeding location at the center, is an extreme over-simplification. A better, but logistically challenging approach would be to determine the true boundaries of any occupied territories by means of radio-tracking and then to measure mole cricket abundance and habitat in the entire territory.

The occurrence of mole crickets depends on soil structure. These insects preferred small-grained soils with lots of sand rather than gravelly soils and soils with limited amounts of sand. They also showed some preference for wet/humid soils. Soil density also influences the occurrence of mole crickets, but the pattern is difficult to interpret. We expected mole crickets to prefer soils of medium density because a soft soil might cause mole cricket galleries to collapse, while in hard soils mole crickets cannot borrow galleries. Yet, we observed a high probability of occurrence in very soft and hard soils. Mole cricket occurrence further declined with increasing vegetation cover. This may be due to a preference of mole crickets for warm soils to ensure an optimal development of their clutches: short, sparse vegetation allows better warming of the soil than dense, high vegetation.

Hoopoe territories clearly differed in quality, as inferred from the non-random pattern of occupancy, relative reproductive output, and from the despotic distribution of male phenotypes. The pattern of territory occupancy and male hierarchical status estimated from phenotypic traits provide spatially-explicit information that might be important for setting conservation priorities: conservation action must focus on those areas in which territories are more frequently occupied (Fig. S1) and that are inhabited by dominant males. These frequently occupied territories produced most of the offspring, and as productivity is an important driver of hoopoe population dynamics [22], these territories are especially important for the survival of this population. Although occupancy data might be easily retrieved, they require a lengthy time span, which may represent a serious handicap in conservation projects. Reliance on more straightforward surrogates of territory occupancy represents a good alternative. As demonstrated here for the hoopoe, useful immediate proxies would be the phenology of territory settlement, the reproductive success achieved in a given territory, as well as the hierarchical status of a male territory holder.

## Supporting Information

Figure S1 **Spatial distribution of territory occupancy.** Map of the study area showing the different territories (black dots). The diameter of the dots corresponds to the number of years a territory was occupied from 2002–2009. For illustration purposes also territories that were never occupied from 2002–2009 are shown (red dots). The Kernel Density Tool of ArcMap 10.1 was used to interpolate the occupancy pattern over the study area. Areas where territories of high occupancy are aggregated are highlighted in white, areas with low occupancy in dark grey. Four large-scale high quality areas can be distinguished.
(JPG)

Table S1 **Descriptive statistics of the continuous habitat variables.** Basic statistical parameters of the different continuous habitat variables considered as predictors for territory quality. Shown are arithmetic means (Mean), standard errors (SE), minimum, maximum and the coefficient of variation (CV) for the radii of 300 m and 200 m. As habitat variables were recorded at 30 sampling points per territory, the statistical parameters shown in the table were calculated from the territory means that were calculated in a first step.
(DOCX)

Table S2 **Influence of parental individual characteristics on breeding success.** Model selection summary of the effects of 1) different morphological traits and 2) different morphological traits plus age on individual breeding success (i.e. number of fledglings) of male and female hoopoes, evaluated by linear mixed effects models. Besides the morphological traits, all models include the following co-variates: year, egg-laying date (of the current brood) and whether the current brood was a first or a second brood. Shown are the differences between the best and the current model (ΔAIC), the AIC weight of the current model (*w*_i_), the number of estimated parameters (*K*), the model deviance and the territory random effect variance (σ^2^).
(DOCX)

Table S3 **Environmental determinants of territory occupancy within tree plantations only.** Model selection summary of the effects of habitat variables, in fruit tree plantations only, on the frequency of territory occupancy within a radius of 300 m (n = 1182) and 200 m (n = 634). Shown are the differences between the best and the current model (ΔAIC), the AIC weight of the current model (*w*_i_), the number of estimated parameters (*K*), the model deviance and the territory random effect variance (σ^2^).
(DOCX)
