# Supplementary figures and images for: Territory Occupancy and Parental Quality as Proxies for Spatial Prioritization of Conservation Areas
Source: PLoS One. 2014 May 16;9(5):e97679. doi: 10.1371/journal.pone.0097679 (PMC4023974; doi:10.1371/journal.pone.0097679)

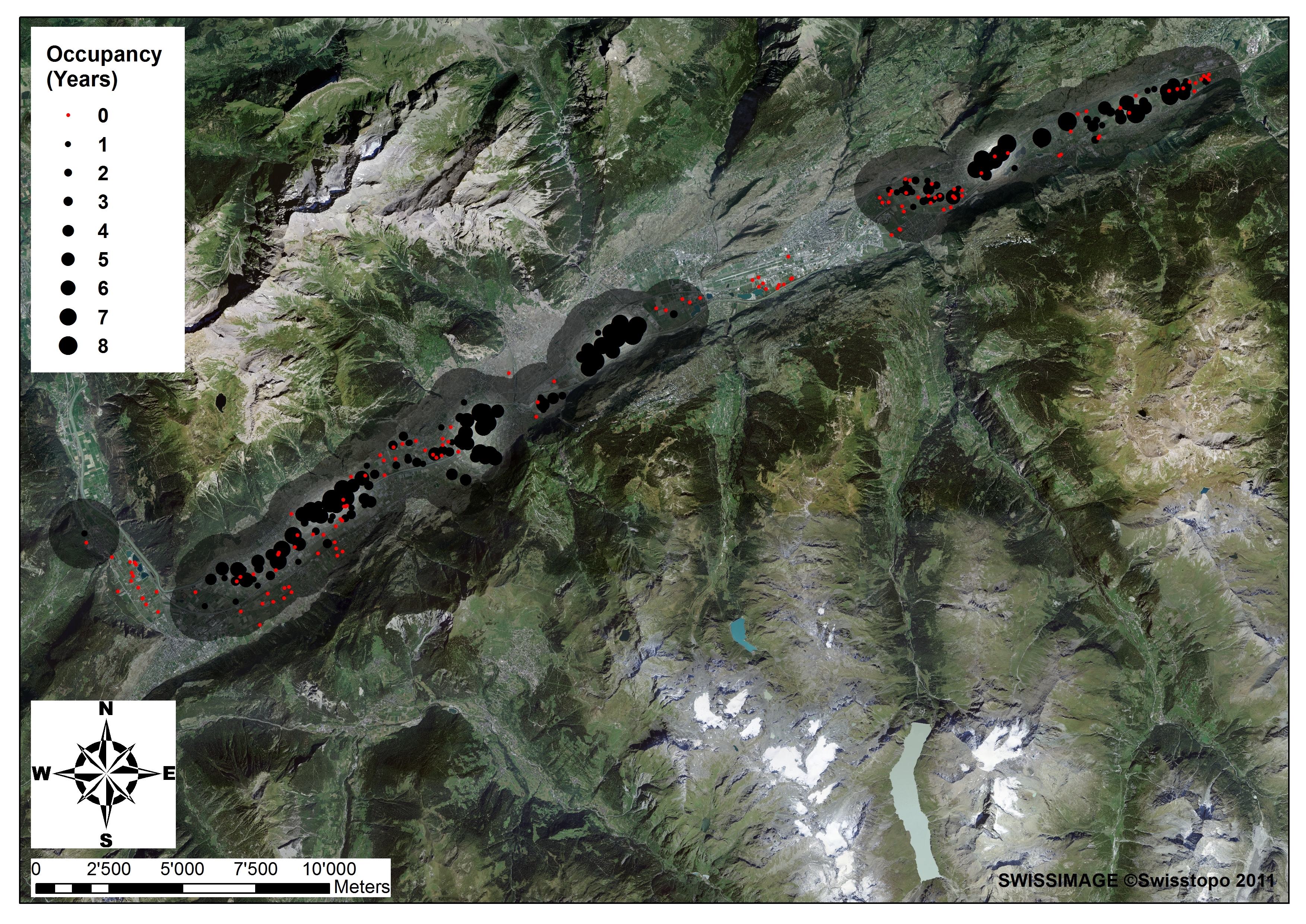

Supplement: Figure S1 — Spatial distribution of territory occupancy. Map of the study area showing the different territories (black dots). The diameter of the dots corresponds to the number of years a territory was occupied from 2002–2009. For illustration purposes also territories that were never occupied from 2002–2009 are shown (red dots). The Kernel Density Tool of ArcMap 10.1 was used to interpolate the occupancy pattern over the study area. Areas where territories of high occupancy are aggregated are highlighted in white, areas with low occupancy in dark grey. Four large-scale high quality areas can be distinguished. (JPG) [file pone.0097679.s001.jpg]
