# Supplementary material for: Territory Occupancy and Parental Quality as Proxies for Spatial Prioritization of Conservation Areas
Source: PLoS One. 2014 May 16;9(5):e97679. doi: 10.1371/journal.pone.0097679 (PMC4023974; doi:10.1371/journal.pone.0097679)
Supplement: Table S1 — Descriptive statistics of the continuous habitat variables. Basic statistical parameters of the different continuous habitat variables considered as predictors for territory quality. Shown are arithmetic means (Mean), standard errors (SE), minimum, maximum and the coefficient of variation (CV) for the radii of 300 m and 200 m. As habitat variables were recorded at 30 sampling points per territory, the statistical parameters shown in the table were calculated from the territory means that were calculated in a first step. (DOCX) [file pone.0097679.s002.docx]

**Table S1. Descriptive statistics of the continuous habitat variables.**

|  | radius = 300 m | | | | | |  | radius = 200 m | | | | | | | |
| --- | --- | --- | --- | --- | --- | --- | --- | --- | --- | --- | --- | --- | --- | --- | --- |
| Model | Mean | SE | Min | Max | | CV |  | Mean | SE | Min | Max | | CV | |  |
| Vegetation cover | 0.72 | 0.01 | 0.41 | | 0.98 | 1.75 |  | 0.71 | 0.02 | 0.19 | | 0.99 | 2.22 |  |  |
| Mole cricket occurrence | 0.86 | 0.01 | 0.75 | | 0.97 | 0.63 |  | 0.85 | 0.01 | 0.60 | | 0.99 | 0.85 |  |  |
| Soil density | 6.89 | 0.19 | 3.85 | | 11.21 | 2.73 |  | 6.67 | 0.22 | 3.62 | | 11.89 | 3.33 | | |

Basic statistical parameters of the different continuous habitat variables considered as predictors for territory quality. Shown are arithmetic means (Mean), standard errors (SE), minimum, maximum and the coefficient of variation (CV) for the radii of 300 m and 200 m. As habitat variables were recorded at 30 sampling points per territory, the statistical parameters shown in the table were calculated from the territory means that were calculated in a first step.
