# Supplementary material for: Territory Occupancy and Parental Quality as Proxies for Spatial Prioritization of Conservation Areas
Source: PLoS One. 2014 May 16;9(5):e97679. doi: 10.1371/journal.pone.0097679 (PMC4023974; doi:10.1371/journal.pone.0097679)
Supplement: Table S2 — Influence of parental individual characteristics on breeding success. Model selection summary of the effects of 1) different morphological traits and 2) different morphological traits plus age on individual breeding success (i.e. number of fledglings) of male and female hoopoes, evaluated by linear mixed effects models. Besides the morphological traits, all models include the following co-variates: year, egg-laying date (of the current brood) and whether the current brood was a first or a second brood. Shown are the differences between the best and the current model (ΔAIC), the AIC weight of the current model (w i), the number of estimated parameters (K), the model deviance and the territory random effect variance (σ2). (DOCX) [file pone.0097679.s003.docx]

**Table S2. Influence of parental individual characteristics on breeding success.**

|  | Males | | | |  | Females | | | |  |
| --- | --- | --- | --- | --- | --- | --- | --- | --- | --- | --- |
| Model | ∆AIC | w_i_ | K | Deviance | σ^2^ | ∆AIC | w_i_ | K | Deviance | σ^2^ |
| 1) Without age |  |  |  |  |  |  |  |  |  |  |
| Intercept | 0.91 | 0.16 | 10 | 1794.8 | 0.00 | 0.00 | 0.20 | 10 | 2076.2 | 0.00 |
| Bill length (mm) | 2.70 | 0.07 | 11 | 1794.6 | 0.00 | 1.78 | 0.08 | 11 | 2076.0 | 0.00 |
| Crest length (mm) | 0.60 | 0.19 | 11 | 1792.5 | 0.00 | 0.63 | 0.15 | 11 | 2074.9 | 0.00 |
| P5 length (mm) | 0.00 | 0.26 | 11 | 1791.9 | 0.00 | 0.15 | 0.19 | 11 | 2074.4 | 0.00 |
| R1 length (mm) | 2.90 | 0.06 | 11 | 1794.8 | 0.00 | 0.79 | 0.14 | 11 | 2075.0 | 0.00 |
| Tarsus length (mm) | 2.90 | 0.06 | 11 | 1794.8 | 0.00 | 0.73 | 0.14 | 11 | 2075.0 | 0.00 |
| Body mass (g) | 0.59 | 0.19 | 11 | 1792.5 | 0.00 | 1.79 | 0.08 | 11 | 2076.0 | 0.00 |
| Bill length + Crest length +  P5 length + R1 length +  Tarsus length + Body mass | 6.52 | 0.01 | 16 | 1788.4 | 0.00 | 5.21 | 0.02 | 16 | 2068.6 | 0.00 |
| 2) Including age |  |  |  |  |  |  |  |  |  |  |
| Intercept | 0.00 | 0.22 | 11 | 1674.3 | 0.00 | 0.05 | 0.19 | 11 | 1885.5 | 0.00 |
| Bill length (mm) | 1.98 | 0.08 | 12 | 1674.3 | 0.00 | 1.89 | 0.08 | 12 | 1885.3 | 0.00 |
| Crest length (mm) | 0.23 | 0.20 | 12 | 1672.5 | 0.00 | 0.00 | 0.20 | 12 | 1883.5 | 0.00 |
| P5 length (mm) | 0.33 | 0.19 | 12 | 1672.6 | 0.00 | 0.05 | 0.19 | 12 | 1883.5 | 0.00 |
| R1 length (mm) | 1.85 | 0.09 | 12 | 1674.1 | 0.00 | 1.58 | 0.09 | 12 | 1885.0 | 0.00 |
| Tarsus length (mm) | 1.95 | 0.08 | 12 | 1674.2 | 0.00 | 0.34 | 0.16 | 12 | 1883.8 | 0.00 |
| Body mass (g) | 1.00 | 0.13 | 12 | 1673.3 | 0.00 | 2.01 | 0.07 | 12 | 1885.5 | 0.00 |
| Bill length + Crest length +  P5 length + R1 length +  Tarsus length + Body mass | 7.92 | 0.00 | 17 | 1670.2 | 0.00 | 4.51 | 0.02 | 17 | 1878.0 | 0.00 |

Model selection summary of the effects of 1) different morphological traits and 2) different morphological traits plus age on individual breeding success (i.e. number of fledglings) of male and female hoopoes, evaluated by linear mixed effects models. Besides the morphological traits, all models include the following co-variates: year, egg-laying date (of the current brood) and whether the current brood was a first or a second brood. Shown are the differences between the best and the current model (∆AIC), the AIC weight of the current model (*w*_i_), the number of estimated parameters (*K*), the model deviance and the territory random effect variance (σ^2^).
