# Supplementary material for: Territory Occupancy and Parental Quality as Proxies for Spatial Prioritization of Conservation Areas
Source: PLoS One. 2014 May 16;9(5):e97679. doi: 10.1371/journal.pone.0097679 (PMC4023974; doi:10.1371/journal.pone.0097679)
Supplement: Table S3 — Environmental determinants of territory occupancy within tree plantations only. Model selection summary of the effects of habitat variables, in fruit tree plantations only, on the frequency of territory occupancy within a radius of 300 m (n = 1182) and 200 m (n = 634). Shown are the differences between the best and the current model (ΔAIC), the AIC weight of the current model (w i), the number of estimated parameters (K), the model deviance and the territory random effect variance (σ2). (DOCX) [file pone.0097679.s004.docx]

**Table S3. Environmental determinants of territory occupancy within tree plantations only.**

|  | radius = 300 m | | | |  |  | radius = 200 m | | | |  |
| --- | --- | --- | --- | --- | --- | --- | --- | --- | --- | --- | --- |
| Model | ∆AIC | *w*_i_ | *K* | Deviance | σ^2^ |  | ∆AIC | *w*_i_ | *K* | Deviance | σ^2^ |
| intercept | 0.00 | 0.34 | 2 | 2877.7 | 3.23 |  | 0.00 | 0.34 | 2 | 1714.5 | 2.73 |
| mow* | 1.94 | 0.13 | 3 | 2877.6 | 3.22 |  | 1.89 | 0.13 | 3 | 1714.4 | 2.72 |
| gw* | 1.97 | 0.13 | 3 | 2877.7 | 3.23 |  | 1.98 | 0.13 | 3 | 1714.5 | 2.72 |
| veg* | 1.98 | 0.13 | 3 | 2877.7 | 3.23 |  | 2.00 | 0.13 | 3 | 1714.5 | 2.73 |
| mole crickets* | 1.99 | 0.13 | 3 | 2877.7 | 3.23 |  | 2.00 | 0.13 | 3 | 1714.5 | 2.73 |
| dens* | 2.00 | 0.13 | 3 | 2877.7 | 3.23 |  | 2.00 | 0.13 | 3 | 1714.5 | 2.73 |
| manag* | 5.90 | 0.02 | 5 | 2877.6 | 3.22 |  | 5.60 | 0.02 | 5 | 1714.1 | 2.70 |
| soilt* | 9.96 | 0.00 | 7 | 2877.7 | 3.23 |  | 9.87 | 0.00 | 7 | 1714.4 | 2.72 |
| dens+gw+veg+mole crickets+soilt+manag+mow* | 25.66 | 0.00 | 15 | 2877.4 | 3.20 |  | 25.34 | 0.00 | 15 | 1713.9 | 2.68 |

Model selection summary of the effects of habitat variables, in fruit tree plantations only, on the frequency of territory occupancy within a radius of 300 m (n = 1182) and 200 m (n = 634). Shown are the differences between the best and the current model (∆AIC), the AIC weight of the current model (*w*_i_), the number of estimated parameters (*K*), the model deviance and the territory random effect variance (σ^2^).

* Covariates: mow = mowing, gw = ground water table, veg = vegetation cover, mole crickets = mole cricket occurrence probability, dens = soil density, manag = ground management, soilt = soil type.
